# Work Engagement and Safety Behavior of Nurses in Specialized Cancer Hospitals: The Mediating Role of Self-Efficacy

**DOI:** 10.1155/2023/9034073

**Published:** 2023-06-15

**Authors:** Fengyan Ma, Yajing Zhu, Lu Liu, Helin Chen, Yan Liu

**Affiliations:** Department of Thoracic Surgery, National Cancer Center/National Clinical Research Center for Cancer/Cancer Hospital, Chinese Academy of Medical Sciences and Peking Union Medical College, Beijing, China

## Abstract

**Aim:**

This paper aimed to examine the current state of nurse safety behavior, and the effect of work engagement on it, and to explore the mediating effect of self-efficacy between work engagement and nurse safety behavior in specialized cancer hospitals.

**Background:**

Nurse safety behavior affects the quality of care and outcomes for patients with cancer. The research proves that self-efficacy is associated with work engagement, but the relationship between self-efficacy, work engagement, and nurse safety behavior needs to be clarified.

**Methods:**

This study used a convenience sampling method to survey 957 nurses from January to February 2023. The survey included tertiary specialized cancer hospitals in five provinces in China. Sociodemography information, the Work Engagement Scale, the Self-Efficacy Scale, and the Nurse Safety Behavior Scale were used for the survey. A Questionnaire Star program collected all the data automatically. The Statistical Package for the Social Sciences version 24 was employed for Pearson correlation and multiple regression analysis to examine the association between work engagement, self-efficacy, and nurse safety behavior. Structural equation models were built using Analysis of Moment Structures version 24.0 software.

**Results:**

The results showed that the nurse safety behavior was at a high level in specialized cancer hospitals. There was a significant positive relationship between the variables work engagement, self-efficacy, and nurse safety behavior with coefficients ranging from 0.244 to 0.564. The results of structural equation modeling revealed that self-efficacy partially mediated the effect between work engagement and nurse safety behavior, accounting for 25.3% of the total effect.

**Conclusion:**

Self-efficacy mediates the relationship between work engagement and nurse safety behavior, and by increasing self-efficacy, nurse safety behavior can be improved, thus ensuring patient safety. *Implications for Nursing Management*. Managers should promote high work engagement and nurse safety behaviors by fostering nurses' self-efficacy.

## 1. Introduction

Patient safety has become the focus of the medical and healthcare industries worldwide. It is the basic principle of medical behavior [1]. It is a risk control process to prevent and reduce risks, errors, and injuries caused to patients in medical procedures [2]. In China, the government and hospital institutions are also placing increasing emphasis on patient safety. In 2018, the notice on further strengthening patient safety management was issued, which for the first time included patient safety in the overall planning of medical quality management and medical institution management. It comprehensively strengthened the construction of patient safety [3].

In 2019, the World Health Organization (WHO) designated 17th September as the World Patient Safety Day. It published “10 facts about patient safety,” noting that adverse events caused by unsafe nursing behaviors are among the top 10 causes of death and disability in the world [4]. In developed countries, one in ten patients are injured by adverse events when receiving hospital treatment and about 50% can be prevented. In low- and middle-income countries, about 134 million adverse events are caused by unsafe care each year, resulting in at least 2.6 million deaths. This demonstrates the importance of nurse safety behavior in promoting patient safety and improving patient outcomes [5].

Nurses have the longest and most frequent contact with patients in clinical work, and their level of safety behavior is related to the implementation of patient safety goals. If the nurse safety behavior decreases, it will have several negative effects, increasing patients' pain, prolonging their hospital stay, and affecting their rehabilitation. It will even affect the overall image of the nursing team and the hospital. Nurses in specialized cancer hospitals care for specific targets. Cancer patients will experience physical symptoms such as pain, fatigue, malnutrition, and severe psychological problems during diagnosis and treatment, such as psychological distress, fear of disease progression, and fear of recurrence, during diagnosis and treatment, which increase their risk of suicide [6, 7]. The negative emotions of cancer patients and the heavy clinical nursing work make nurses more prone to compassion fatigue, job burnout, and even resignation intentions, which can easily cause potential safety hazards and increase the incidence of adverse events [8, 9]. Therefore, it is important to investigate the nurse safety behavior in specialized cancer hospitals.

Current clinical research has shown that nurse safety behavior is influenced by many factors such as patients' perceptions of safety culture, safety leadership, safety performance, and nursing work environment [10–12]. Studies in the construction industry have shown that employees who are actively involved in their work have higher levels of safety behavior [13]. However, there are no studies on work engagement and nurse safety behavior in healthcare. Nurses who receive a better deal for their work are more engaged, which improves not only their performance but also their overall productivity and quality of care [14]. This suggests that high levels of work engagement may have a positive impact on nurse safety behavior.

There is a paucity of research on nurses' work engagement in specialized cancer hospitals, focusing mainly on studies that examine the factors that influence it and its relationship with variables such as structural empowerment and self-efficacy. One of these studies suggests that self-efficacy can positively influence nurses' work engagement [15]. Research has also shown that nurses' self-efficacy in specialized cancer hospitals can strengthen their communication skills, and professional values, and effectively reduce their burnout, and psychological distress, resulting in higher levels of safety behavior [16–18]. Therefore, we hypothesized that self-efficacy may mediate the relationship between work engagement and nurse safety behavior.

In summary, this study aimed to examine the relationship between work engagement and nurse safety behavior. The mediation effect model verified the mediating effect of self-efficacy between work engagement and nurse safety behavior. To provide a clinical reference for hospital administrators to formulate and implement measures for nurse safety behavior, reduce nursing errors and deficiencies, ensure nursing safety, and improve nursing quality.

### 1.1. Theoretical Framework and Hypotheses

#### 1.1.1. Nurse Safety Behavior

Nurse safety behavior refers to the behavior state of nursing staff when performing nursing tasks for patients, including compliance with the nursing code of conduct and core system, and implementation of safety nursing measures [19]. This is an essential component of ensuring patient safety, improving nursing quality management, and preventing adverse events [20].

#### 1.1.2. Work Engagement and Nurse Safety Behavior

Work engagement is a positive and work-related emotional and cognitive state of caregivers characterized by vitality, dedication, and concentration [21]. Vitality means that nurses are not afraid of difficulties and can voluntarily participate in work in a complete working state. Dedication means that nurses have a strong sense of meaning and pride in their work, as well as full enthusiasm for the work, and the courage to face the challenges in work. Concentration means that the nurse is totally absorbed in the creation and takes pleasure in it.

This study is based on Bakker's work demand-resource model [22]. The model suggests that work engagement depends on the balance between work demands and resources. Work must require the individual to make continuous physical, psychological, cognitive, and emotional efforts to complete all aspects of the work. Nurses with high levels of work engagement have a positive emotional and mental state and can communicate better with patients [23]. They will be full of energy at work, and their physical strength will be easy to recover. They will have high safety behavior and provide better quality care to patients to improve patient safety and promote patient recovery. Work resources can offset the negative effects of work demand, stimulate individual work enthusiasm, improve work participation, and produce good work outcomes. Therefore, the hypotheses of the study were as follows:

  H1. Work engagement has a positive impact on nurse safety behavior

#### 1.1.3. The Mediating Role of Self-Efficacy

The essence of the work demand-resource model is that the internal motivation and external conditions work together, with external conditions driving the individual's internal motivation. Self-efficacy is an individual's ability to complete a task, which creates internal motivation and plays an important role in an individual's cognition and behavior [24, 25]. Nurses' self-efficacy mainly refers to their ability to realize self-value in nursing and to be competent in nursing work. Research shows that the higher nurses' self-efficacy, the more enthusiasm, and energy they can put into their work, maintain a positive work attitude, take the initiative to undertake work, complete nursing work effectively, and improve patient safety [26]. Mache et al. verified the correlation between self-efficacy and work engagement and pointed out that self-efficacy is an essential resource of personal psychology that can complement the physical and mental consumption of nurses required by work and reduce burnout [26]. Nurses' self-efficacy can improve work pressure and job burnout, improve work engagement, and thus improve nursing safety behavior, ultimately providing high-quality nursing services for patients, and promoting patient safety and health [27, 28]. Therefore, the hypotheses of the study were as follows:

  H2. Self-efficacy can improve nurse safety behavior in specialized cancer hospitals
  H3. The variable of self-efficacy mediates the relationship between work engagement and nurse safety behavior

## 2. Methods

### 2.1. Study Design

A cross-sectional design was used.

### 2.2. Participants and Settings

Nurses from tertiary specialized cancer hospitals in five Chinese provinces were selected to participate in the survey from January to February 2023 using a convenience sampling method. Inclusion criteria were as follows: (i) age ≥ 18 years; (ii) postclinical nurses who have obtained registered nurse qualification certificates; and (iii) informed consent and voluntary participation in this study. Exclusion criteria were as follows: (i) nurses on maternity leave or sick leave and (ii) nurses in postgraduate study and practice.

The sample size for this study was calculated based on the principle that the questionnaire items were 10 to 20 times the main research variable. A total of 31 questionnaire items were designed for this study, taking into account 20% of invalid questionnaires, so a sample size of between 372 and 744 was required. The final sample consisted of 957 cases that met the requirements.

### 2.3. Variables and Instruments

A general data questionnaire is designed by consulting previous research literature, including gender, age, educational level, job position, title, employment form, years of service, monthly income, marital status, weekly working hours, the total number of adverse events experienced in career, and safety training courses attended.

The Utrecht Work Engagement Scale (UWES) was developed by Schaufeli et al. [29] in 2006 to simplify the original (UWES-17), resulting in a simplified version of the 9-item version (UWES-9). The Chinese version was translated and back-translated by Chinese scholar Li et al. [30]. The scale consists of three-dimensions, namely, vitality, dedication, and focus, with a total of nine items. The Liken 7-point scale was used, with scores ranging from 0 to 6 on a scale from “never” to “always,” with higher scores indicating higher levels of work engagement. The internal consistency coefficient of the total scale in this study was 0.946, and the Cronbach's alpha coefficient of each dimension is 0.864, 0.907, and 0.854, respectively.

The General Self-Efficacy Scale (GSES) was initially prepared by Schwarzer and Born [31] and translated and revised by Wang et al. [32]. The scale has a total of 10 items and is a single-dimensional scale. Likert's four-level scoring method is used. The score ranges from 1 to 4 points, from “completely wrong” to “completely right.” A higher score is associated with a greater sense of self-efficacy. In this study, the Cronbach's alpha coefficient for the scale was 0.889.

The Nurse Safety Behavior Questionnaire (NSBQ) was developed by Shih et al. [33] and translated into Chinese by Rong [34]. The questionnaire is one-dimensional with 12 items. It is scored on a five-point Likert scale, with 1 being “never” and 5 being “always.” The higher the score, the better the nurse's performance in patient safety behavior. The Cronbach's alpha coefficient of the scale in this study is 0.899.

### 2.4. Pilot Study

A pilot study was conducted to assess the clarity and time required to complete the questionnaire. In the pilot study, 50 nurses from the participating hospitals were recruited for a pre-test, and these nurses were then excluded from the study sample. Nurses were instructed to understand the content of the scale and to complete the questionnaire, which took approximately 15–20 minutes to complete. The results showed that the questionnaire had high reliability and validity and met the study criteria.

### 2.5. Procedure

This study was approved prior to data collection. The researcher then obtained the appropriate hospital consent by contacting the nursing department of the hospital concerned. The online questionnaire Star platform was used to distribute and collect the questionnaire. The head nurse of the department was contacted to explain the purpose and significance of this survey, and who and how to complete it. The matron then distributed the questionnaire to the department's WeChat group with a deadline of 5 days for submission. The first page of the questionnaire was written in a consistent language to explain the purpose, method, and requirements for completing the survey. Once the questionnaire was developed, each IP only had to complete it once and the questions were presented in a flip page format. Subjects could not submit until all questions had been answered online. The study was voluntary. At the end of the survey, two researchers checked the questionnaires submitted by the respondents, one by one, and immediately eliminated incomplete questionnaires. A total of 1100 questionnaires were distributed and 1023 questionnaires were returned. After excluding 20 questionnaires with all the same answers and 23 questionnaires with incomplete information, the number of valid questionnaires was 957, with a valid return rate of 93%.

### 2.6. Data Statistics

The data were analyzed using IBM SPSS v24.0 and Amos 24.0. To describe the general information of clinical nurses and the current situation of safety behavior, the count data were expressed as frequency and composition ratio, and all data were tested for normal distribution, the measurement data were expressed as mean ± standard deviation. A validation factor analysis was carried out and Cronbach's alpha was calculated to ensure the reliability of the scale items used in this study. Pearson correlation analysis was used to analyze the correlation between the variables. The multiple regression analysis was performed with NSBQ as the dependent variable and two statistically significant variables (UWES and GSES) in the correlation analysis as independent variables. First, based on the results of Pearson's correlation analysis, AMOS 24.0 software was applied to construct a mediating effects model to explore the relationship among work engagement, self-efficacy, and nurse safety behavior of specialized cancer hospitals. In addition, the maximum likelihood ratio method was used to fit the data and modify the model. The model fit indices and suggested cut-off values were examined for results including incremental fit index (IFI > 0.8), goodness-of-fit index (GFI > 0.8), adjusted goodness-of-fit (AGFI > 0.8), comparative fit indices (CFI > 0.90), and root mean square error of approximation (RMSEA < 0.08). Finally, bootstrapping was performed using 5000 bootstrap samples and 95% bias-corrected confidence intervals (CI) to test the significance of direct and indirect effects [35]. *P* < 0.05 was considered to be a statistically significant difference.

### 2.7. Ethical Consideration

According to Chinese law and institutional guidelines, no ethical review was required for this study. The study strictly adhered to institutional requirements and ethical standards, participants were informed of the purpose and use of the study during the examination process, informed consent was obtained, and participation was voluntary. The study was conducted anonymously to ensure that nurses' privacy was not violated. In addition, the study is an anonymous investigation that does not involve unethical behavior or human clinical trials and does not result in any adverse health consequences, physical or mental, for the participants.

## 3. Results

### 3.1. Descriptive Statistics of Sociodemography Information

A total of 957 nurses from specialized cancer hospitals, as shown in Table 1. The majority of participants were female (94.6%) and married (62.3%). 72.4% had a bachelor's degree in nursing and had a mean of 9 years of experience with a standard deviation of 8 years. The mean age of the sample population was 32 years, with a standard deviation of 7 years. Of the respondents (*n* = 957), the majority of the study participants were employed on a contract basis (75.5%) and had a monthly income between 5,000 and 10,000 yuan (60.5%). In addition, Table 1 shows that the majority of the study participants had experienced no more than 10 total adverse events during their careers (94.7%), and most had received safety training (94.7%).

### 3.2. Reliability and Validity

Confirmatory factor analysis was used to verify the measures that were used. First, the Kaiser–Meyer–Olkin (KMO) test and Bartlett's sphericity test were used to test the adequacy of sampling. The results showed that the KMO and Bartlett's sphericity test values for the nurse safety behavior scale were 0.935 (*P* < 0.001), for the work engagement scale 0.925 (*P* < 0.001), and for the self-efficacy scale 0.925 (*P* < 0.001). The factor loadings for all the constructs used in this study were greater than the recommended value of 0.7 [36], indicating support for the construct validity of the scales. Reliability was also analyzed to verify that the items of the scales met Cronbach's alpha. The results of the study showed that the Cronbach's values for all scales and their dimensions ranged from 0.854 to 0.946, meaning that the overall internal consistency reliability of the scales was high.

### 3.3. Work Engagement, Self-Efficacy, and Nurse Safety Behavior Score in the Specialized Cancer Hospitals

In the specialized cancer hospital, the nurse work engagement score was (47.81 ± 10.62) at a moderate level. The self-efficacy score was (31.30 ± 14.65), which was mild to high. The safety behavior score was (57.31 ± 4.41) at a high level (Table 1).

### 3.4. Correlation Study

Pearson correlation analysis showed that work engagement was positively correlated with self-efficacy (*r* = 0.564 and *P* < 0.001). Nurse safety behavior was weakly positively correlated with work engagement and self-efficacy (*r* = 0.264, *r* = 0.244 and *P* < 0.001) (Table 2).

### 3.5. Regression Analysis of Nurse Safety Behavior

The multiple regression analysis was performed with NSBQ as the dependent variable and two statistically significant variables (UWES and GSES) in the correlation analysis as independent variables. The Adjusted *R*^2^ value was 0.081, showing that the UWES and GSES models explained 8.1% of the variance in the NSBQ (Table 3).

### 3.6. A Test of the Hypothesized Model Is Conducted

**Figure 1:**
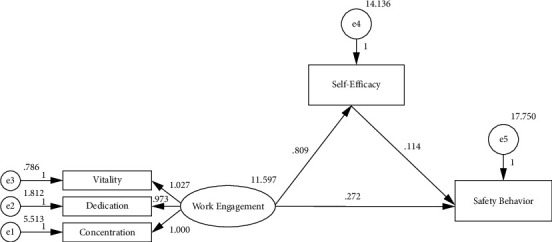
Fitting model of the mediating effect of self-efficacy.

The model was fitted, validated, and modified using the maximum likelihood ratio method with AMOS version 24.0. As a result, the following results were obtained: *X*^2^/d*f* = 2.592, GFI = 0.996, AGFI = 0.984, CFI = 0.998, IFI = 0.998, and RMSEA = 0.071. According to the model fit index (Figure 1), the model was acceptable. The mediating effect was investigated using a Bootstrap sampling test. The results showed that for the effect of work engagement on nurse safety behavior, 5000 random samples were repeated from the original data. Mediating effects of nurses' self-efficacy in specialized cancer hospitals had a 95% confidence interval of 0.032 to 0.153, excluding 0, which was statistically significant (*P* < 0.01). The findings indicate that self-efficacy mediates the effect and accounts for 25.3% of the total effect (Table 4).

## 4. Discussion

This study explores the current status of nurse safety behavior in the specialized cancer hospital and the relationship between nurses' self-efficacy, work engagement, and safety behavior. The detailed results were as follows: the nurse safety behavior score in the specialized cancer hospital was at a high level. Work engagement is a positive predictor of nurse safety behavior. Self-efficacy plays a part-mediating role between nurses' work engagement and nurse safety behavior.

As an important participant in the provision of medical services, nurses' safety behavior can ensure patient safety, improve the quality of care and prevent the occurrence of adverse events. The results of this study show that the nurse safety behavior score in specialized cancer hospitals is (57.31 ± 4.41) points, which is at a high level, higher than the psychiatric nurse safety behavior score (47.98 ± 7.45) points [37], and the nurse safety behavior score in the emergency department of tertiary hospitals is 55 (49,58) points [38]. In recent years, the Chinese government has promoted the safety and quality management of medical institutions [39], all medical institutions have actively carried out the implementation of quality control and the construction of a patient safety culture, which has improved the safety awareness of nurses to a certain extent and made them show higher safety behaviors [10]. Many studies have shown that the construction of a patient safety culture improves nurse safety behavior, patient safety, and medical quality and affects patient outcomes [40–42]. Cancer patients have physical and psychological problems such as pain, fatigue, and fear of disease progression, recurrence, and prognosis, which lead to potential safety hazards of self-injury and suicide [6, 7, 43]. Nurses will improve risk awareness and increase safety behavior when facing cancer patients. Nurses will face occupational exposure to radiation and antineoplastic drug when nursing cancer patients, increasing their awareness of self-protection and improving safety behavior. However, in the face of cancer patients and high occupational exposure risks, nurses in specialized cancer hospitals will bear more pressure, even if they show vital safety behaviors, resulting in job burnout and even resignation intention [44, 45].

This study shows that the nurse work engagement score in the specialized cancer hospital is (47.81 ± 10.62), which is at the middle level and is similar to the result of Xu's nurse work engagement score in China's cancer hospitals [15], which indicates that nurse work engagement in specialized cancer hospitals needs to be improved. This study is the first to verify that the higher the degree of engagement of nurses, the higher their safety behavior, which is consistent with the research results of Saleem et al. [13] within the construction industry, and Wang and Lu [46] in the coal industry. Work engagement is a positive, happy, work-related emotion and cognitive state characterized by vitality, dedication, and focus, which reflects the sense of identity and focus in work [21]. Work input can improve nurses' satisfaction and job burnout, increase their willingness to stay on duty [47, 48], and improve nurses' work results and quality [49]. Nurses in specialized cancer hospitals have a moderate level of work engagement, reducing their safety behavior to a certain extent. This may be due to the negative impact of heavy work, compassion fatigue, occupational exposure risk, and other adverse effects on their work enthusiasm and professional identity, which has seriously affected the degree of work engagement, leading to a decline in the level of safety behavior [50, 51].

The structural equation model shows that nurses' self-efficacy in specialized cancer hospitals can directly and positively affect work engagement and safety behavior. Self-efficacy plays a partial intermediary role in the impact of work engagement on safety behavior, and the intermediary effect value accounts for 25.3% of the total effect. Self-efficacy is an individual's belief in his ability to complete tasks, which plays a role in controlling and regulating behavior. It plays a vital role in an individual's cognition and behavior [24, 25]. In the work demand-resource model, work demand is the internal factor of individual work engagement. Several studies have shown that [24, 25, 52] a higher sense of self-efficacy can stimulate nurses to have the internal drive to meet challenges, overcome difficulties, and better complete tasks, and can enable nurses to devote themselves to nursing work with a positive attitude and dedication. Work resource is the external factor of individual work engagement. The research shows that [53–56] external factors such as a good working environment, beneficial medical and nursing cooperation, and high sense of motivation make them more actively and diligently participate in nursing work. Self-efficacy, as the internal driving force in the creation of nurses, can make them more actively participate in safety training and acquire safety knowledge, which can be reflected in their behavior. A higher sense of self-efficacy can improve nurses' ability to cope with work pressure and occupational risks and improve their safety behavior. This study validates that nurses' self-efficacy is a way to translate high levels of work engagement into nurse safety behavior.

## 5. Limitations

This study used a convenience sampling method by posting the link to the online questionnaire via WeChat. Although questionnaires were collected from several hospitals, the total number of nurses in each specialist oncology hospital was not surveyed beforehand, thus conducting quota sampling and stratified sampling, resulting in an uneven sample size of hospitals and weakening the representativeness of the data. This study was a cross-sectional study, and the results showed a weak correlation between the coefficient of correlation between nurse safety behavior and work engagement and self-efficacy, and the low variability explained in the regression analysis affected the stability of the model. However, the influence between the three was positive. Future studies should attempt to include more influential factors to fully explore the factors influencing nurse safety behavior and to conduct longitudinal studies to further validate them.

## 6. Conclusions

This study explains how developing nurses' self-efficacy is an intervention mechanism that promotes their work engagement and enhances nurse safety behavior. Self-efficacy and nurse work engagement improve safety behavior, and self-efficacy partially mediates work engagement and safety behavior among nurses in specialized cancer hospitals.

## 7. Implications for Nursing Management

Nursing managers should focus on cultivating nurses' self-efficacy. They can improve nurse safety behavior, reduce nursing errors and defects, and improve nursing quality by creating a good working environment, establishing a healthy healthcare partnership, increasing nurses' sense of professional interest, and appropriately empowering nurses to mobilize their motivation and professional identity so that they can devote themselves to nursing work and increase their work participation. We can improve nurse safety behavior by exploring new safety management models, strengthening a diverse patient safety culture, creating a safe environment, creating a safe atmosphere, and formulating safe production processes.

This study is significant as it has a solid theoretical basis in proposing relationships between research variables. In addition, this study provides ideas for care managers to effectively control patient safety.

## Figures and Tables

**Table 1:** Sociodemography information of participant nurses (*N* = 957).

Characteristics	Categories	Mean ± SD or *n* (%)
Gender	Male	52 (5.4)
	Female	905 (94.6)

Age (years)		32.00 ± 7.90
Education level	Junior college	138 (14.4)
	Adult college entrance examination	114 (11.9)
	Undergraduate	693 (72.4)
	≥Postgraduate	12 (1.3)

Job position	None	843 (88.1)
	Tutor	71 (7.4)
	Head nurse	43 (4.5)

Title	Nurse	242 (25.3)
	Nurse practitioner	367 (38.3)
	Supervisor nurse	315 (32.9)
	≥Deputy chief nurse	33 (3.4)

Years of service		9.94 ± 8.44
Employment form	Formal incorporation	149 (15.6)
	Contract employment	723 (75.5)
	Personnel agency	85 (8.9)

Monthly income	1 k–5 k	142 (14.8)
	5 k–10 k	579 (60.5)
	10 k–15 k	199 (20.8)
	15 k–20 k	35 (3.7)
	>20 k	2 (0.2)

Marital status	Married	596 (62.3)
	Single	361 (37.7)

Weekly working hours	≤40	712 (74.4)
	>40	245 (25.6)

Total number of adverse events experienced in career	<10	886 (92.6)
	10–50	48 (5.0)
	>50	5 (0.5)
	Unknown	17 (1.8)

Safety training courses	Attended	906 (94.7)
	Not-attended	51 (5.3)

Work engagement		47.81 ± 10.62
Self-efficacy		31.30 ± 4.65
Nurse safety behavior		57.31 ± 4.41

**Table 2:** Association analysis of work engagement, self-efficacy, and nurse safety behavior in specialized cancer hospitals (*n* = 957).

Variable	Work engagement	Self-efficacy	Nurse safety behavior
Work engagement	1	—	—
Self-efficacy	0.564^*∗∗*^	1	—
Nurse safety behavior	0.264^*∗∗*^	0.244^*∗∗*^	1

^*∗∗*^ *P* < 0.001.

**Table 3:** Regression analysis of nurse safety behavior (*n* = 957).

Variable	*B*	SE	*β*	*t*	*P*
Norm	141.983	9.791	—	14.502	<0.001
Work engagement	0.077	0.016	0.186	4.954	<0.001
Self-efficacy	0.132	0.036	0.139	3.707	<0.001

*R* ^2^ = 0.083, adjusted *R*^2^ = 0.081, *F* = 43.251, *P* < 0.001.

**Table 4:** Moderating effect of self-efficacy between work engagement and safety behavior of nurses in specialized cancer hospitals (*n* = 957).

Effects	Structural paths	Impact	SE	*P*	95% CI	%
Direct effects	UWES ⟶ NSBQ	0.272	0.055	<0.001	0.174–0.389	74.7
Mediating effects	UWES ⟶ GSES ⟶ NSBQ	0.092	0.031	0.003	0.032–0.153	25.3
Total effects	UWES ⟶ NSBQ	0.364	0.049	<0.001	0.277–0.174	

*Note.* UWES = work engagement, GSES = self-efficacy, NSBQ = nurse safety behavior.

## Data Availability

The data used to support the findings of this study are available from the corresponding author upon request.
